# Characterization of the Oncogenic Potential of Eukaryotic Initiation Factor 4A1 in Lung Adenocarcinoma via Cell Cycle Regulation and Immune Microenvironment Reprogramming

**DOI:** 10.3390/biology11070975

**Published:** 2022-06-28

**Authors:** Kuan-Li Wu, Yung-Chi Huang, Yu-Yuan Wu, Chao-Yuan Chang, Yung-Yun Chang, Hung-Hsing Chiang, Lian-Xiu Liu, Ying-Ming Tsai, Jen-Yu Hung

**Affiliations:** 1 Graduate Institute of Medicine, College of Medicine, Kaohsiung Medical University, Kaohsiung 807, Taiwan; 980448kmuh@gmail.com (K.-L.W.); beryl1992@gmail.com (Y.-C.H.); chaoyuah@kmu.edu.tw (C.-Y.C.); ji394122@gmail.com (L.-X.L.); 2 Division of Pulmonary and Critical Care Medicine, Kaohsiung Medical University Hospital, Kaohsiung 807, Taiwan; cyy807@gmail.com (Y.-Y.C.); yingming@kmu.edu.tw (Y.-M.T.); 3 School of Medicine, College of Medicine, Kaohsiung Medical University, Kaohsiung 807, Taiwan; fred901229@gmail.com; 4 Department of Anatomy, College of Medicine, Kaohsiung Medical University, Kaohsiung 807, Taiwan; 5 Division of General Medicine, Kaohsiung Medical University Hospital, Kaohsiung 807, Taiwan; 6 Division of Thoracic Surgery, Department of Surgery, Kaohsiung Medical University Hospital, Kaohsiung Medical University, Kaohsiung 807, Taiwan; shiiiiidae@gmail.com; 7 Drug Development and Value Creation Research Center, Kaohsiung Medical University, Kaohsiung 807, Taiwan; 8 Department of Internal Medicine, Kaohsiung Municipal Ta-Tung Hospital, Kaohsiung 807, Taiwan

**Keywords:** cell cycle, DNA repair, *EIF4A1*, LUAD, tumor immune microenvironment

## Abstract

**Simple Summary:**

Lung cancer is a common cancer throughout the world. Despite advanced treatment strategies, the outcome is still poor. Based on the results of the present study, *EIF4A1* interacting with EIF4H manipulates cell cycle regulation and immune microenvironment reprogramming in lung adenocarcinoma. The results specify *EIF4A1* in lung adenocarcinoma tumorigenesis. Treatment derived from *EIF4A1* would be worthy of further investigation.

**Abstract:**

Lung adenocarcinoma (LUAD) is a common type of lung cancer. Although the diagnosis and treatment of LUAD have significantly improved in recent decades, the survival for advanced LUAD is still poor. It is necessary to identify more targets for developing potential agents against LUAD. This study explored the dysregulation of translation initiation factors, specifically eukaryotic initiation factors 4A1 (*EIF4A1*) and *EIF4A2*, in developing LUAD, as well as their underlying mechanisms. We found that the expression of *EIF4A1*, but not *EIF4A2*, was higher in tumor tissue and associated with poor clinical outcomes in LUAD patients. Elevated expression of EIF4H with poor prognosis may potentiate the oncogenic role of *EIF4A1*. Functional enrichment analysis revealed that upregulation of *EIF4A1* was related to cell cycle regulation and DNA repair. The oncogenic effect of *EIF4A1* was further elucidated by Gene Set Variation Analysis (GSVA). The GSVA score of the gene set positively correlated with *EIF4A1* was higher in tumors and significantly associated with worse survival. In the meantime, gene set enrichment analysis (GSEA) also indicated that elevated *EIF4A1* expression in LUAD patients was associated with a decreased infiltration score for immune cells by reducing anticancer immune cell types and recruiting immunosuppressive cells. Consistent with the results, the GSVA score of genes whose expression was negatively correlated with *EIF4A1* was lower in the tumor tissue of LUAD cases with worse clinical outcomes and was strongly associated with the disequilibrium of anti-cancer immunity by recruiting anticancer immune cells. Based on the results from the present study, we hypothesize that the dysregulation of *EIF4A1* might be involved in the pathophysiology of LUAD development by promoting cancer growth and changing the tumor immune microenvironment. This can be used to develop potential diagnostic biomarkers or therapeutic targets for LUAD.

## 1. Introduction

Lung cancer, a dreadful cancer, has topped the list of cancer-related deaths worldwide for decades. Recently, the development of novel agents, such as targeted therapies and immunotherapies, has improved the survival of lung cancer patients. However, the five-year survival rate for stage IV lung cancer patients is still lower than ten percent. Nowadays, modern technologies, such as next-generation sequencing (NGS) [1], databases, such as cancer genomics programs, and bioinformatics have been utilized to discover novel genes that mediate tumorigenesis, improve cancer diagnosis and predict the survival of cancer patients [2]. Despite the progress in exploring novel genes and their targeted agents, the outcome for lung cancer patients is still poor.

Translating proteins from mRNAs requires three steps: initiation, elongation, and termination [3,4]. The initiation step has been closely associated with the organogenesis and prognosis of many types of human cancers [5]. Two members of the *EIF4A* (eukaryotic initiation factor 4A) family in mammals play a critical role in initiation: *EIF4A1* and *EIF4A2* [6]. They also serve as archetypal members of the DEAD-box family [7], as *EIF4A1* (DDX2A), *EIF4A2* (DDX2B), and *EIF4A3* (DDX48) [8]. *EIF4A1* is more abundant in the cytoplasm than *EIF4A2*. *EIF4A1* and *EIF4A2* are closely linked in the initiation step of translation.

*EIF4A1* and *EIF4A2*, highly similar cytoplasmic proteins, show approximately 90% sequence identity [9]. As the archetypal member of the *EIF4A* family, *EIF4A1* was first identified for its necessity in translation [10,11]. It has bidirectional RNA helicase functions and acts as an RNA-dependent ATPase [12]. Genome-wide studies have suggested that *EIF4A*-dependent mRNAs are associated with cell proliferation, cell survival, cell cycle progression and angiogenesis through activation of the PI3K and RAS pathways [13,14]. Aberrant expression patterns of the *EIF4A* family’s genes have been found in different tumors [15,16,17]. Moreover, *EIF4A* family genes have been closely correlated with immune cell infiltration in different cancers [18]. They may be related to the dysfunction of the RNA helicase, which results in the expression of proteins produced by abnormal RNA translation [19].

Reducing cancer deaths will require further identification of the genes involved in cancer development and the tumor microenvironment. Such gene information will help inform the development of actionable drugs. In this study, we took advantage of powerful LUAD NGS data and bioinformatics tools to determine whether *EIF4A1* and *EIF4A2* expression profiles are correlated with LUAD. Based on the results of the present study, it appears that *EIF4A1* may be a useful marker in studying the progression of LUAD.

## 2. Materials and Methods

### 2.1. Data Collection

The tissues of adjacent non-tumor lungs and tumors were harvested from the Division of Thoracic Surgery and Division of Pulmonary and Critical Care Medicine, Kaohsiung Medical University Hospital (Kaohsiung, Taiwan, KMUH-IRB-20130054; KMUH-IRB-20180023). All patients signed the informed consent form. The gene expression quantification datasets of LUAD were extracted from samples of TCGA (The Cancer Genome Atlas. Available online: https://portal.gdc.cancer.gov, accessed on 15 March 2022) [20]. The criterion in the analysis was *p*-value < 0.05, which was calculated using UALCAN (The University of Alabama at Birmingham CANcer data analysis Portal. Available online: http://ualcan.path.uab.edu, accessed on 15 March 2022) [21]. The genes correlated with *EIF4A1*, either positively or negatively, were also extracted from UALCAN. The lung tissue protein expression was extracted from the Human Protein Atlas website (Available online: https://www.proteinatlas.org/ENSG00000161960-*EIF4A1*/pathology, accessed on 26 March 2022) [22].

### 2.2. Survival Analysis of EIF4A1 and EIF4H Using KM Plotter

The survival analyses of the candidate genes in LUAD were assessed via the KM plotter (The Kaplan Meier plotter. Available online: http://kmplot.com/analysis/, accessed on 19 March 2022) [23]. The Kaplan–Meier plotter is designed to assess the correlation between the expression of specific genes and different types of survival. Patients were divided into two groups, with the best cut-off computed for the best discrimination of median survival between groups. The hazard ratios (95% confidence intervals) were calculated using the Cox proportional model.

### 2.3. DNA Methylation and Copy Number

The extent of *EIF4A1* DNA methylation was compared with respect to the tissue source, the tumor stages, and the stages of lymph node metastasis via the UALCAN website [21]. The copy number variation (CNV) of *EIF4A1* in patients with LUAD was extracted from the TCGA Pan-Cancer (PANCAN) dataset from the UCSC Xena website (The University of California, Santa Cruz. Available online, https://xena.ucsc.edu/, accessed on 20 March 2022) [24]. Pearson’s correlation between the *EIF4A1* mRNA expression level and the copy number and/or DNA methylation was calculated using the metadata.

### 2.4. Screening for Differentially Expressed miRNAs

The regulation of *EIF4A1* by candidate microRNAs (miRs) was predicted via TargetScan (Available online: https://www.targetscan.org, accessed on 1 April 2022) [25], which searched the target genes based on the conserved sites matching the seed regions of miRNAs.

### 2.5. Functional Analysis

CancerSEA was used to elucidate the functions of *EIF4A1* (CancerSEA: a cancer single-cell state atlas. Available online: http://biocc.hrbmu.edu.cn/CancerSEA/home.jsp, accessed on 5 April 2022) [26]. Gene set enrichment analysis (GSEA) is a computational tool that evaluates whether a priori-defined gene set presents statistically significant and concordant differences between two biological or pathological states. To investigate the role of *EIF4A1*, the LUAD patients of TCGA were divided into *EIF4A1* high-expressed and low-expressed groups according to the highest and lowest quartiles, and GSEA was conducted to analyze the enrichment of datasets between high- and low-*EIF4A1* groups. False discovery rate (FDR) < 0.05 and nominal *p*-value < 0.05 were set as the cutoff criteria. The gene set “c2.cp.kegg.v6.2.symbols.gmt” was chosen as the reference gene set.

### 2.6. The GSVA of Gene Sets

The correlation of the gene sets positively or negatively correlated with *EIF4A1* was also extracted from UALCAN. The criteria in the analysis were Pearson-CC (correlation coefficient) > 0.3 and *p*-value < 0.05, which was calculated using UALCAN. The GSVA score of the gene sets with regards to gene expression, survival rate, and immune infiltration was calculated using GSCA (Gene Set Cancer Analysis. Available online: http://bioinfo.life.hust.edu.cn/GSCA/#/, accessed on 19 April 2022) [27].

### 2.7. Signaling Pathway Analysis

The pathway analysis of the gene sets positively and negatively correlated with *EIF4A1* was assessed using Ingenuity Pathway Analysis software (IPA, QIAGEN Digital Insights, Redwood City, CA, USA). The final number of genes analyzed using the IPA software was 161, and 44 mRNAs with positive or negative correlation with *EIF4A1* were also analyzed. The significant enrichment analysis of the two groups of gene sets was also assessed based on the Kyoto Encyclopedia of Genes and Genomes (KEGG) using the Database for Annotation, Visualization and Integrated Discovery (DAVID) (Available online: https://david.ncifcrf.gov/, accessed on 22 March 2022) [28], a tool for functional annotation analysis. A *p*-value < 0.05 was considered significant.

### 2.8. Statistical Analyses

All statistical analyses were conducted using Prism (Version 9.0.2). The Student’s *t*-test was used for statistical comparisons. Spearman’s correlation was applied for the analysis of the correlation. A *p*-value < 0.05 was regarded as statistically significant.

## 3. Results

### 3.1. The Upregulated Expression of EIF4A1 Genes in Lung Adenocarcinoma (LUAD)

**Figure 1:**
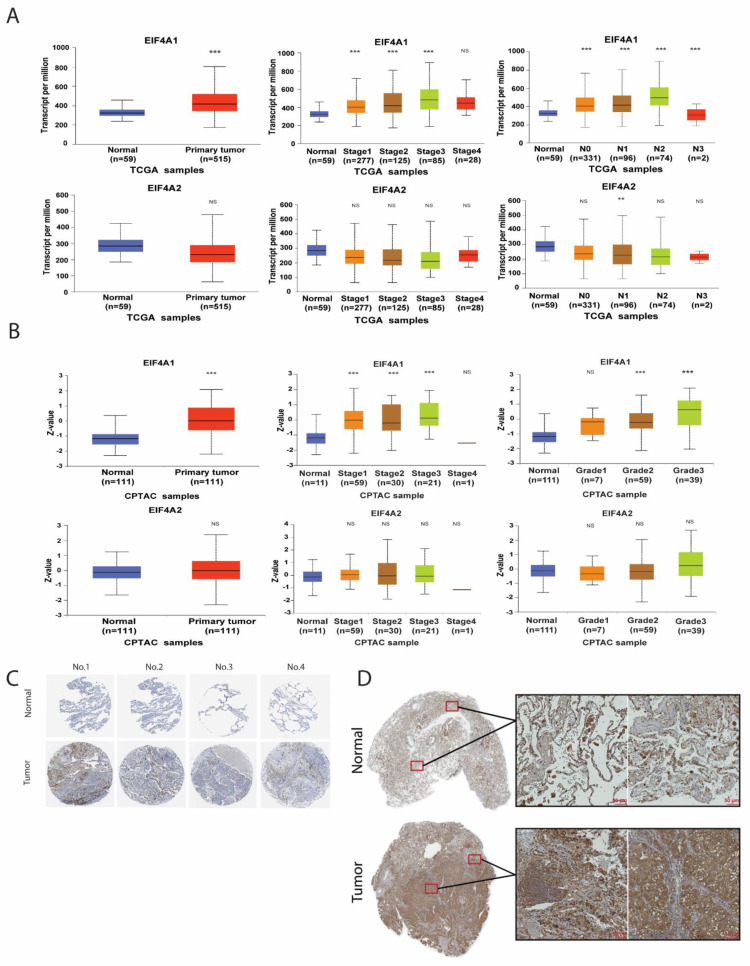
The expression of the *EIF4A* family at mRNA and protein levels in LUAD. The gene expression levels of *EIF4A1* and *EIF4A2* from the TCGA LUAD cohort are shown: normal vs. tumor (**left panel**, **A**); by stage (**middle panel**, **A**); and by lymph node status (**right panel**, **A**). Further, the protein levels of *EIF4A1* and *EIF4A2* from the CPTAC cohort are demonstrated: normal vs. tumor (**left panel**, **B**); by stage (**middle panel**, **B**); and by lymph node status (**right panel**, **B**). The IHC staining of *EIF4A1* in a normal lung and in LUAD from the Human Protein Atlas (**C**) and our in-house cohort (**D**). **: *p* < 0.01; ***: *p* < 0.005; NS: not significant.

To identify if *EIF4A1* and *EIF4A2* expression were correlated with lung cancer development, we assessed the expression of both genes in normal and LUAD tumor tissue using a TCGA cohort. The expression level of *EIF4A1* in tumor tissues was higher than that in normal tissue samples of LUAD, but this was not the case for the expression of *EIF4A2* (left panel, Figure 1A), and the expression levels were higher in the advanced stages and advanced lymph node status for *EIF4A1* but not *EIF4A2* (right two panels, Figure 1A). However, the upregulated expression of *EIF4A1* was neither lymph node status-dependent nor stage-dependent (upper right two panels, Figure 1A). We further validated the protein levels of *EIF4A1* and *EIF4A2* using the CPTAC database on the UALCAN website. Similarly, higher protein levels of *EIF4A1* but not *EIF4A2* (left panel, Figure 1B) were detected in the tumor tissues of LUAD. The expression levels of *EIF4A1* were tumor grade-dependent (right two panels, Figure 1B) but not concordant with individual cancer stages. In contrast, the protein levels of *EIF4A2* (right two panels, Figure 1B) did not change as tumor grades or stages changed. Moreover, immunohistochemical staining (IHC) data extracted from the Human Protein Atlas showed that *EIF4A* levels were greater in tumors than in normal tissue (Figure 1C). In addition, our in-house cohort had the same result (Figure 1D). These results suggest that both *EIF4A1* mRNA and protein expression are elevated in LUAD.

### 3.2. Elevated Levels of EIF4A1 Confer Poor Survival

**Figure 2:**
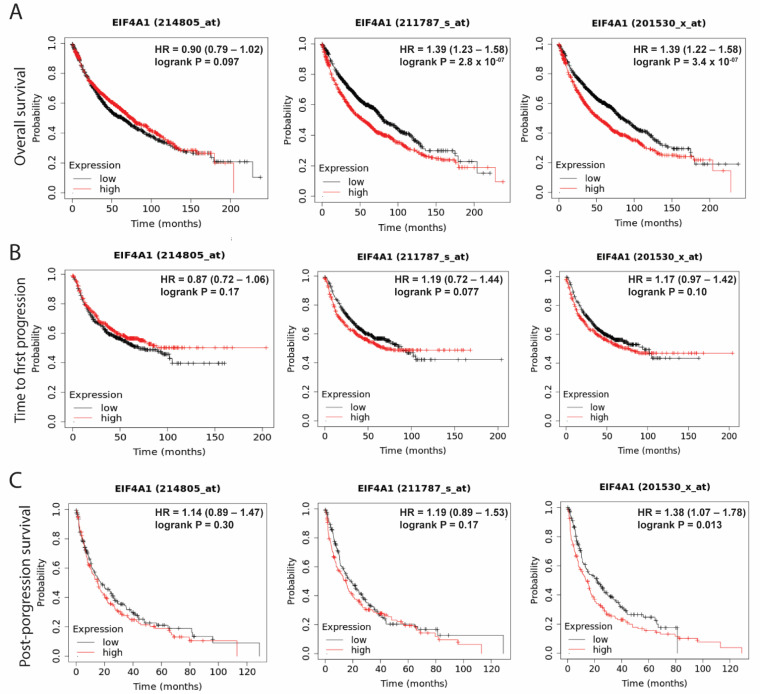
*EIF4A1* is associated with a poor prognosis in LUAD. The association of *EIF4A1* expression and survival time through data extracted from the KM plotter website on overall survival disadvantage when expression is high with divergent trends (*p* < 0.05) (**A**), time to first progression (**B**) and post-progression survival (**C**). Red and black lines: high- and low-expressed *EIF4A1*, respectively. Each dot: a censored patient. HR: hazard ratio, that is, high-expressed hazard rate/low-expressed hazard rate; an HR over 1 means a survival disadvantage.

The clinical significance of *EIF4A1* was evaluated using a survival analysis from the Kaplan–Meier plotter (the correlation between gene expressions and survival). Higher expression levels of *EIF4A1* were linked to shorter overall survival, which was demonstrated by two out of three probes for *EIF4A1* (Figure 2A). On the contrary, the *EIF4A1* level was not associated with the time to first progression (Figure 2B). Regarding post-progression survival, an elevated *EIF4A1* level was linked to shorter survival in one out of three probes (Figure 2C). The data suggest that higher *EIF4A1* expression in a tumor may confer a survival disadvantage.

### 3.3. The Epigenetic Regulatory Mechanisms for EIF4A1 Expression

**Figure 3:**
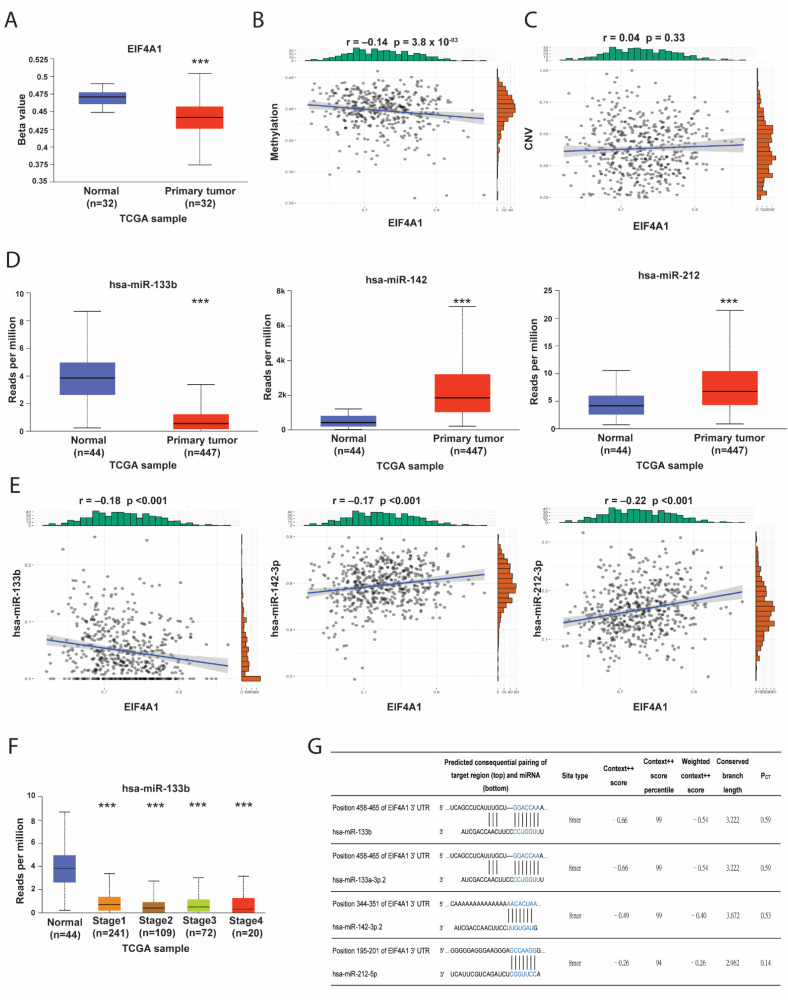
miR-133b could be a regulator of *EIF4A1* in LUAD. We investigated the epigenetic regulatory mechanisms in terms of DNA methylation with regards to correlation and copy number variation. The low expression levels of *EIF4A1* methylation in tumor tissues compared with normal ones (**A**) and its negative correlation (**B**). The expression correlation between DNA copy number and *EIF4A1* (**C**). In addition, tissue levels of the predicted miRNAs (miR-133b, miR-142 and miR-212) of *EIF4A1* from TargetScan in normal vs. tumor tissues (**D**). The correlations between the predicted miRNAs and *EIF4A1* (**E**), and hsa-miR-133b expression correlated with stages (**F**). The predicted binding sites of miR-133b in the 3′UTR of *EIF4A1* mRNA and its context++ score (**G**). ***: *p* < 0.005.

Gene expression can be regulated by epigenetic and post-translational modifications. We found DNA methylation levels to be lower in the tumor tissues and that they had an insignificantly negative correlation (r = −0.14) with *EIF4A1* (Figure 3A,B). The copy number of *EIF4A1* was not concordant with its mRNA expression level (Figure 3C), precluding DNA methylation and copy number variation (CNV) as the primary regulatory mechanisms of *EIF4A1*. The miR–RNA interaction predicted by TargetScan suggested that hsa-miR-133b, hsa-miR-142 and hsa-miR-212 were possible candidates. Among these, hsa-miR-133b was expressed in low levels in tumor tissues compared with normal tissues (Figure 3D). Moreover, the negative correlation between *EIF4A1* and the curated miRs was only observed for hsa-miR-133b, but not for hsa-miR-142 and hsa-miR-212 (Figure 3E). The expression of hsa-miR-133b was also low in advanced tumor stages without any dependency on the stages (Figure 3F). The binding score for hsa-miR-133b on *EIF4A1* was high (99%) (Figure 3G). These results suggest that hsa-miR-133b is a regulator of *EIF4A1* in LUAD.

### 3.4. The Co-Operation of EIF4H with EIF4A1 in LUAD

**Figure 4:**
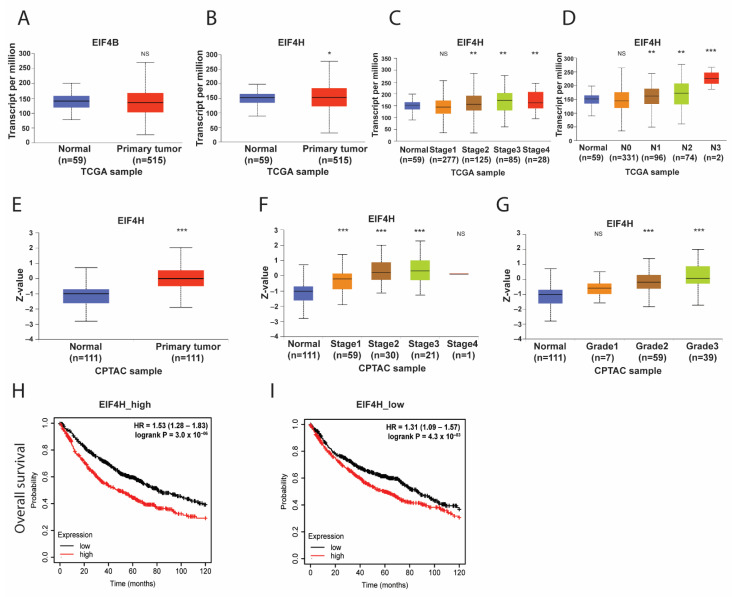
The interaction of *EIF4A1* with *EIF4H* may affect the OS of LUAD patients. The expressions of *EIF4B* (**A**) and *EIF4H* (**B**) at mRNA levels in the normal and tumor tissues of LUAD patients. The *EIF4H* mRNA expression in tumor staging (**C**) and lymph node status (**D**). The EIF4H protein expression in normal and tumor tissues (**E**), tumor staging (**F**) and lymph node status (**G**). The cross-analysis of *EIF4A1* and OS in *EIF4H*-high (**H**) and *EIF4H*-low (**I**) LUAD patients. *: *p* < 0.05; **: *p* < 0.01; ***: *p* < 0.005; NS: not significant.

Because the activity of *EIF4A* is regulated by two homologous RNA-binding proteins, EIF4B and EIF4H [29], we assessed the influences of EIF4B and EIF4H on *EIF4A* in LUAD. As shown in Figure 4A,B, the expression of *EIF4H* at mRNA levels, but not *EIF4B*, was enhanced in the tumor tissue of patients with LUAD. The increased expression of the *EIF4H* gene was also positively correlated with advanced clinical stages and lymph node status (Figure 4C,D). The enhancement of EIF4H at protein expression was also observed in the tumors of LUAD patients (Figure 4E). Similarly, positive correlations between EIF4H protein and higher clinical stages or lymph node status were also illustrated (Figure 4F,G). Cross-analysis of *EIF4A1–EIF4H* in OS time showed that the hazard ratio (HR) of up-regulated *EIF4A1* on OS declined from 1.53 (*p* = 3 × 10^−6^) to 1.31 (*p* = 0.0043) across LUAD patients with higher and lower *EIF4H*, respectively (Figure 4H,I). These data suggest that the interaction of *EIF4A1* and *EIF4H* could affect the clinical outcome in LUAD patients.

### 3.5. The Upregulation of EIF4A1 Contributes to the Regulation of the Cell Cycle and Tumor Microenvironment

**Figure 5:**
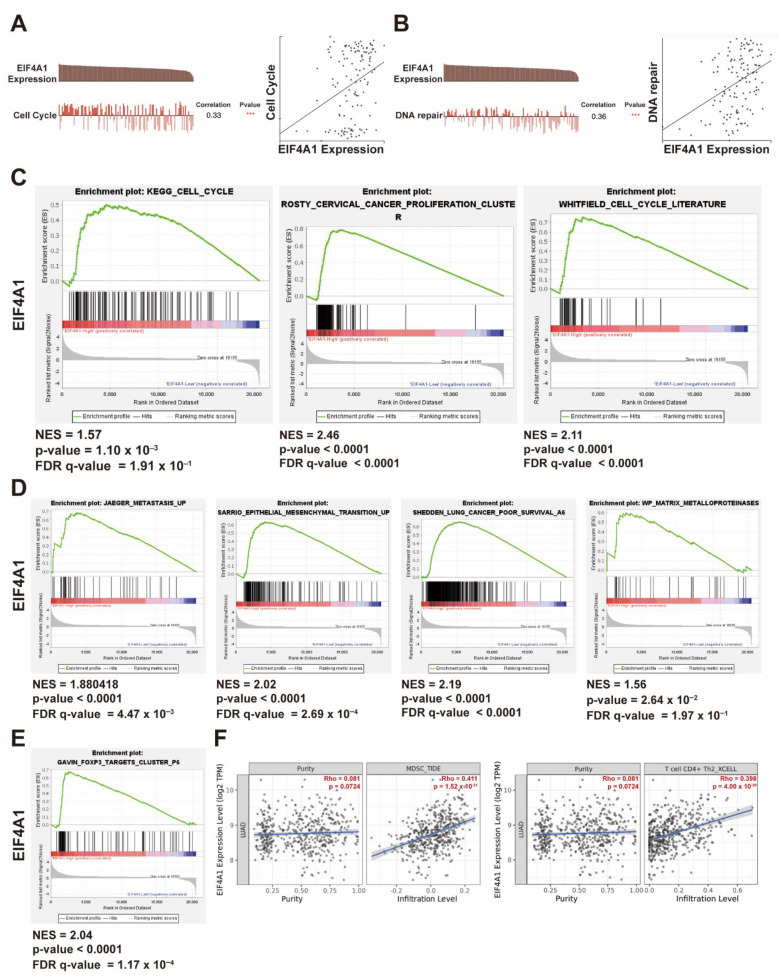
Elevated *EIF4A1* expression is associated with cancer growth and progression. *EIF4A1* is involved in regulating the cell cycle (**A**) and DNA repair (**B**), as indicated by the CancerSEA website. The transcriptomes of LUAD patients with high *EIF4A1* expression are associated with cell cycle and cancer proliferation (**C**), metastasis, epithelial-to-mesenchymal transition (EMT) and poor clinical outcomes (**D**). The gene set of the *FOXP3* target, as analyzed by GSEA, is correlated with high *EIF4A1* expression (**E**). Positive correlations of *EIF4A1* with MDSC and Th2 T cell infiltration (**F**). ***: *p* < 0.005.

To assess the biologic function of *EIF4A1*, we performed functional analysis using the public database CancerSEA. The results revealed that *EIF4A1* was related to the cell cycle (r = 0.33) and DNA repair (r = 0.36) in LUAD (Figure 5A,B). We also divided LUAD patients in the TCGA cohort into *EIF4A1* high- and low-expression groups, and then applied the grouped gene expression profiles onto GSEA analysis. The results showed that *EIF4A1* and its regulated gene sets were mainly involved in cell cycle progression and cancer proliferation (Figure 5C). Matrix metalloproteinase (MMP), cancer metastasis and poor prognosis were also associated with elevated *EIF4A1* expression in LUAD patients (Figure 5D). Interestingly, FOXP3, a marker of regulatory T (Treg) cells, is also strongly associated with high *EIF4A1* expression (Figure 5E). A comprehensive resource for the systematical analysis of immune infiltrates, that is, the TIMER2.0 website, also indicated that the expression of *EIF4A1* was positively correlated with the infiltration of myeloid-derived suppressor cells (MDSC) and Th2 CD4^+^ T cells (Figure 5F).

### 3.6. The Gene Set Positively Correlated with EIF4A1 Controls Cancer Growth and Progression via Cell Cycle Regulation and Immunity Suppression

**Figure 6:**
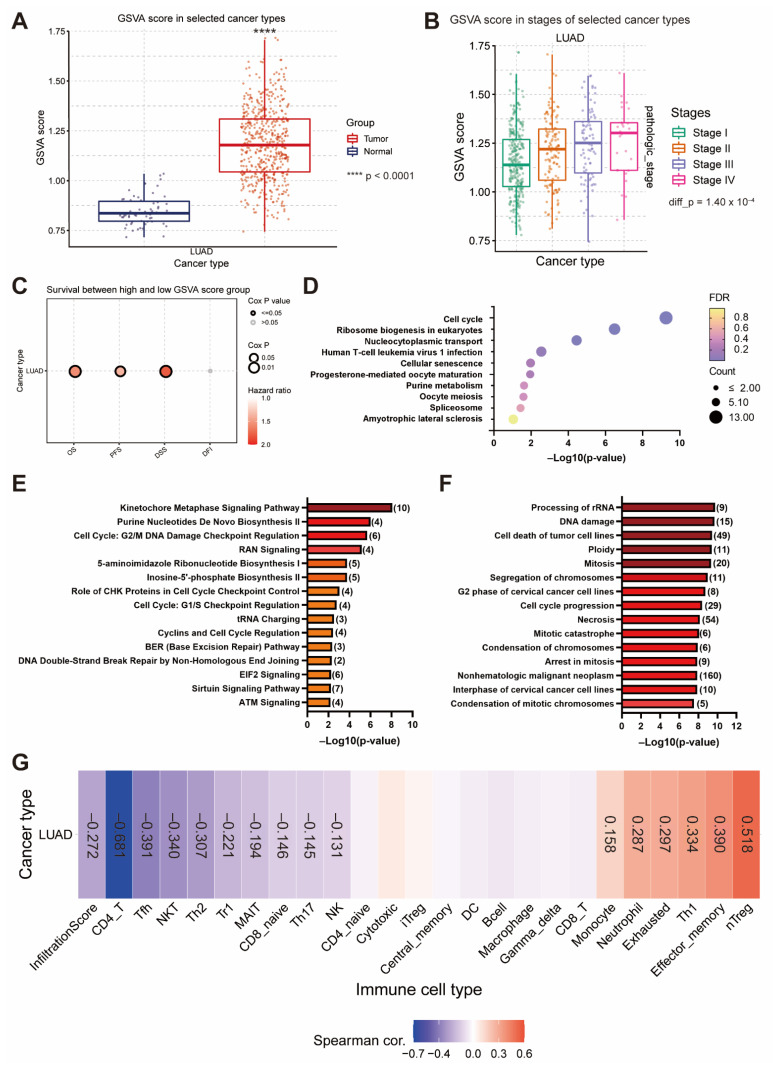
The gene sets positively correlated with *EIF4A1* contribute to cancer cell growth and immunity suppression. The GSVA score of the gene set positively correlated with *EIF4A1*, sub-grouped by normal and tumor tissues (**A**) and clinical stages (**B**). The survival associations using different outcome indicators in high- or low-GSVA score patients of LUAD (**C**). KEGG pathway of the gene set positively correlated with *EIF4A1* (**D**). Pathway analysis of the gene set positively correlated with *EIF4A1* in the canonical pathway (**E**) and disease and function (**F**), as assessed via IPA. The correlation of immune cell infiltration with the GSVA score of the gene set positively correlated with *EIF4A1* (**G**). ****: *p* < 0.001.

To elucidate the signaling network of *EIF4A1* in cancer development, we extracted the gene sets with a positive correlation to *EIF4A1* in LUAD. We found that 161 genes (r > 0.4, *p* < 0.05) were strongly associated with *EIF4A1* (Table S1). The GSVA score, calculated via pathway enrichment analysis using the pre-defined gene set, was higher in tumor tissues than in normal tissues among LUAD patients (Figure 6A). The GSVA score also correlated positively with tumor stage (I to IV) in LUAD (*p*-value = 0.0001) (Figure 6B). The higher GSVA score of this gene set was linked to poor prognosis, including shorter OS (HR = 1.57, *p*-value = 0.002), PFS (HR = 1.37, *p*-value = 0.01) and disease-specific survival (DSS) (HR = 1.80, *p*-value = 0.0002) (Figure 6C). Consistent with GSEA analysis, KEGG pathway analysis using a transcriptome of high-*EIF4A1*-expressing tumors revealed that the gene sets were involved in cell cycle regulation (Figure 6D). IPA analysis of both the canonical pathway and disease and function also supported that the gene set was associated with cell cycle- and DNA repair ATM signaling (Figure 6E,F). The influence of the immune microenvironment associated with this gene set and positively correlated with *EIF4A1* was also analyzed. We found a negative association with immune cell infiltration but increased recruitment of immunosuppressive cells (exhausted and nTreg) (Figure 6G).

### 3.7. The Gene Set Negatively Correlated with EIF4A1 Modulates Antigen-Presenting and Anticancer Immune Cell Infiltration

**Figure 7:**
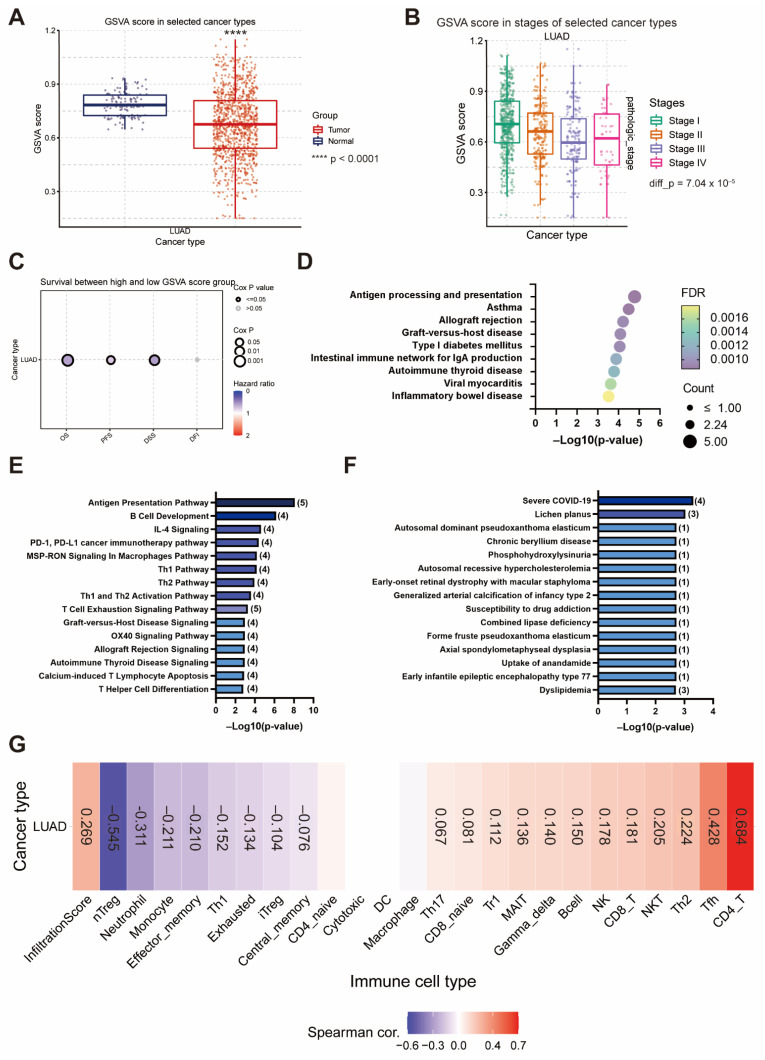
The gene sets negatively correlated with *EIF4A1* hamper cancer development and enhance immunity. The GSVA score of the gene set negatively correlated with *EIF4A1*, sub-grouped by normal and tumor tissues (**A**) and clinical stages (**B**). The survival associations using different outcome indicators in high- or low-GSVA score patients of LUAD (**C**). KEGG pathway of the gene set negatively correlated with *EIF4A1* (**D**). Pathway analysis of the gene set negatively correlated with *EIF4A1* in the canonical pathway (**E**) and diseased and function (**F**), as assessed via IPA. The correlation of immune cell infiltration with the GSVA score of the gene set negatively correlated with *EIF4A1* (**G**). ****: *p* < 0.001.

To elucidate the signaling networks suppressed by *EIF4A1* upregulation, we also assessed the gene set negatively correlated with *EIF4A1*. The results showed that 44 genes (r < −0.3, *p* < 0.05) were negatively associated with *EIF4A1* expression in the TCGA LUAD cohort (Table S2). The GSVA score of the gene set negatively correlated with *EIF4A1* was lower in the tumor tissues (0.673 vs. 0.787, *p* < 0.0001) (Figure 7A). The GSVA scores decreased at stage I and then gradually decreased at stage III (Figure 7B). The prognosis analysis also indicated that a higher GSVA score in these downregulated gene sets was linked to a better prognosis, including longer OS (HR = 0.60, *p* = 0.0007), PFS (HR = 0.77, *p* = 0.039) and DSS (HR = 0.57, *p* = 0.004) (Figure 7C). KEGG pathway analysis revealed several immune-related pathways, such as “Antigen processing and presentation”, which was the action network of this downregulated gene set (Figure 7D). IPA analysis for the canonical pathway also supported that the gene set was associated with the “Antigen Presentation Pathway”, while several immune activation statuses, such as “Infectious Diseases”, were modulated by this downregulated gene set (Figure 7E,F). Moreover, in contrast to the upregulated gene sets, GSVA analysis of this gene set also demonstrated a positive correlation with immune cell infiltration and the abundancy of various anti-cancer immune cells (CD8_naive, Tr1, Th2, Th17, Tfh, NKT, MAIT, B cell, NK, γ-δ, CD4_T and CD8_T cells). On the contrary, immunosuppressive cells (exhausted, nTreg and iTreg) were inversely associated with the gene set (Figure 7G).

## 4. Discussion

Lung cancer has been one of the most deadly malignancies worldwide for decades. However, the current treatment strategies for lung cancer remain unsatisfactory, especially in patients without driver mutations [30]. Our study indicates that *EIF4A1* acts as an oncogene in lung cancer development. Clinically, LUAD patients with high levels of *EIF4A1* carry poor outcomes. Furthermore, *EIF4A1* potentiates oncogenesis in LUAD by regulating the cell cycle and by modulating the tumor immune microenvironment. This study provides evidence that *EIF4A1* is a potential target for developing therapeutic agents for LUAD.

Growing evidence indicates that translation dysregulation is an important step contributing to cancer development and progression [31]. Translation initiation is the rate-limiting step of mRNA translation. It has been proposed that cancer cells become ‘addicted’ due to the increased capacity of cancer cells to cope with metabolic stress and maintain cancer growth [18,32]. *EIF4A1* has been reported to directly determine the selective translation of oncoproteins, such as myc, myb, notch, cdk6, bcl-2 and ROCK1, which are critical regulators contributing to cancer survival, proliferation, migration, invasion, metastasis and chemoresistance [14,33,34]. Targeting the translation initiation components is considered a potential strategy for therapeutic interventions against cancer [32,35]. The therapeutic potential of the *EIF4A* inhibitor eFT226 has been investigated in B cell lymphoma and Burkitt lymphoma models through the coordinated translational inhibition of oncogenic drivers and transcription factors [35]. In this study, we found that the expression of *EIF4A1* was increased in tumors of LUAD compared with normal tissues, although the relationships were neither lymph node metastasis- nor stage-dependent. Notably, the survival analyses from the KM plotter revealed lower overall survival time (OS) and post-progression survival (PPS) but not time to first progression (FP) in lung cancer patients with high *EIF4A1* expression as well as expression of its correlated genes. Functional analysis of all 161 genes positively correlated with *EIF4A1* revealed that *EIF4A1*-related genes facilitated cancer growth by regulating cell cycle progression. This was also further supported by CancerSEA and GSEA analyses of the transcriptomes of LUAD patients with higher levels of *EIF4A1*. These results indicate that *EIF4A1* might have an oncogenic function in LUAD by influencing cell division. *EIF4A1* helicase activity is required for the pathogenic function of *EIF4A1* by synergizing *EIF4A* to unwind secondary structures or facilitate the translation of a subset of oncogenic mRNAs [36]. The helicase activity of *EIF4A1* is enhanced by EIF4H, which can interact with *EIF4A* by increasing the affinity of *EIF4A* for RNA [37]. Reportedly, *EIF4H* is overexpressed in various cancers, such as colorectal cancer and glioma [36,38]. The splicing factor, RNA binding motif protein 10 (RBM10), suppresses LUAD progression by regulating alternative splicing of *EIF4H* exon 5 [39]. In our study, the expression of *EIF4H* was upregulated in the tumor tissues of LUAD patients who had poor clinical outcomes. Cross-analysis of *EIF4A1* and *EIF4H* demonstrated that the negative impact of *EIF4A1* on overall survival was lessened in patients with lower *EIF4H* expression, indicating that the enhancement of *EIFH* was required for the oncogenic potential of this translational initiation factor *EIF4A1* in LUAD.

The microenvironment of lung cancer becomes immunosuppressive due to the regulation of the immune response activated by malignant cells, which aids tumor cells in escaping from immune surveillance [40,41]. Due to genetic instability, constant tumor cell division changes their phenotypes toward a poor immunogenicity that can avoid immune attack [42,43]. In addition, cancer cells recruit FOXP3^+^ regulatory T cells (Tregs), Th2 T cells and myeloid-derived suppressor cells (MDSCs) and trigger the exhaustion of T cells, which play a more dominant role compared to cytotoxic immune cells, such as effector CD8^+^ T cells, NK or NKT cells and γδ T cell or effector CD4^+^ T cells, during cancer progression. This results in a dampening of the antitumor immune response and the facilitation of tumor progression [44,45]. In our study, we found via GSEA analysis that LUAD patients with higher *EIF4A1* had increases in the FOXP3^+^ singling cascade, and we also found via website prediction that MDSC and Th2 cells would infiltrate. The gene set positively correlated with *EIF4A1* was strongly associated with reduced infiltration of anti-cancer immune cells such as NKT/NK cells and CD4^+^ T cells, and this accompanied a reduced infiltration score. In contrast, the gene set negatively correlated with *EIF4A1* was related to increased recruitment of NK/NKT cells, γδ T cells and CD4^+^ T cells, resulting in an increased infiltration score. Taken together, our findings indicate that *EIF4A1* may reprogram the tumor immune microenvironment via decreased immunogenicity and immunosuppressive cell recruitment. However, further experimental verification is needed to validate our predictions.

## 5. Conclusions

To sum up, this study identified that *EIF4A1* is differentially expressed in tumor and non-tumor tissues of LUAD patients. Simultaneously, GSEA confirmed the pathogenic role of *EIF4A1* in LUAD in promoting cell cycle progression and remodeling the tumor immune microenvironment. Bioinformatics approaches identified *EIF4A1* and its partner *EIF4H* as possible LUAD marker genes, providing ideas for other experimental studies in the future. However, further study is mandatory to understand more about *EIF4A1*’s activities and its potential impact as a biomarker or therapeutic target for LUAD therapy.

## Data Availability

CancerSEA, available online: http://biocc.hrbmu.edu.cn/CancerSEA/home.jsp, accessed on 5 April 2022; DAVID (Database for Annotation, Visualization and Integrated Discovery), available online: https://david.ncifcrf.gov/, accessed on 22 March 2022; GSCA (Gene Set Cancer Analysis, available online: http://bioinfo.life.hust.edu.cn/GSCA/#/, accessed on 19 April 2022); the Human Protein Atlas website avalible online: https://www.proteinatlas.org/ENSG00000161960-*EIF4A1*/pathology, accessed on 26 March 2022; K–M plotter, available online: http://kmplot.com/analysis/, accessed on 19 March 2022; TargetScan, available online: https://www.targetscan.org, accessed on 1 April 2022; TCGA (The Cancer Genome Atlas), available online: https://portal.gdc.cancer.gov, accessed on 15 March 2022; UALCAN (The University of Alabama at Birmingham CANcer data analysis Portal), available online: http://ualcan.path.uab.edu, accessed on 15 March 2022); UCSC Xena website, available online, https://xena.ucsc.edu/, accessed on 20 March 2022.

## Supplementary Materials

The following supporting information can be downloaded at https://www.mdpi.com/article/10.3390/biology11070975/s1: Table S1: The gene lists of positive correlation with *EIF4A1*; Table S2: The gene lists of negative correlation with *EIF4A1*; and Table S3: TCGA cases and accession number.
