# Supplementary material for: Characterization of the Oncogenic Potential of Eukaryotic Initiation Factor 4A1 in Lung Adenocarcinoma via Cell Cycle Regulation and Immune Microenvironment Reprogramming
Source: Biology (Basel). 2022 Jun 28;11(7):975. doi: 10.3390/biology11070975 (PMC9311917; doi:10.3390/biology11070975)
Supplement: Supplementary file 1 [file biology-11-00975-s001.zip › biology-1772343-supplementary-Table S3.pdf]

Table S3. TCGA cases and accession number.

Normal	Tumor								
TCGA-38-4625-11	TCGA-05-4244-01	TCGA-44-3398-01	TCGA-49-AARE-01	TCGA-55-6982-01	TCGA-55-8619-01	TCGA-69-7979-01	TCGA-78-7536-01	TCGA-91-7771-01	TCGA-J2-A4AE-01
TCGA-38-4626-11	TCGA-05-4249-01	TCGA-44-3918-01	TCGA-49-AARN-01	TCGA-55-6983-01	TCGA-55-8620-01	TCGA-69-7980-01	TCGA-78-7537-01	TCGA-91-8496-01	TCGA-J2-A4AG-01
TCGA-38-4627-11	TCGA-05-4250-01	TCGA-44-3919-01	TCGA-49-AARO-01	TCGA-55-6984-01	TCGA-55-8621-01	TCGA-69-8253-01	TCGA-78-7539-01	TCGA-91-8497-01	TCGA-L4-A4E5-01
TCGA-38-4632-11	TCGA-05-4382-01	TCGA-44-4112-01	TCGA-49-AARQ-01	TCGA-55-6985-01	TCGA-55-A48X-01	TCGA-69-8254-01	TCGA-78-7540-01	TCGA-91-8499-01	TCGA-L4-A4E6-01
TCGA-44-2655-11	TCGA-05-4384-01	TCGA-44-5643-01	TCGA-49-AARR-01	TCGA-55-6986-01	TCGA-55-A48Y-01	TCGA-69-8255-01	TCGA-78-7542-01	TCGA-91-A4BC-01	TCGA-L9-A443-01
TCGA-44-2657-11	TCGA-05-4389-01	TCGA-44-5644-01	TCGA-4B-A93V-01	TCGA-55-6987-01	TCGA-55-A48Z-01	TCGA-69-8453-01	TCGA-78-7633-01	TCGA-91-A4BD-01	TCGA-L9-A444-01
TCGA-44-2661-11	TCGA-05-4390-01	TCGA-44-5645-01	TCGA-50-5044-01	TCGA-55-7227-01	TCGA-55-A490-01	TCGA-69-A59K-01	TCGA-78-8640-01	TCGA-93-7347-01	TCGA-L9-A50W-01
TCGA-44-2662-11	TCGA-05-4395-01	TCGA-44-6145-01	TCGA-50-5045-01	TCGA-55-7281-01	TCGA-55-A491-01	TCGA-71-6725-01	TCGA-78-8648-01	TCGA-93-7348-01	TCGA-L9-A5IP-01
TCGA-44-2665-11	TCGA-05-4396-01	TCGA-44-6146-01	TCGA-50-5049-01	TCGA-55-7283-01	TCGA-55-A492-01	TCGA-71-8520-01	TCGA-78-8655-01	TCGA-93-8067-01	TCGA-L9-A743-01
TCGA-44-2668-11	TCGA-05-4397-01	TCGA-44-6147-01	TCGA-50-5051-01	TCGA-55-7284-01	TCGA-55-A493-01	TCGA-73-4658-01	TCGA-78-8660-01	TCGA-93-A4JN-01	TCGA-L9-A7SV-01
TCGA-44-3396-11	TCGA-05-4398-01	TCGA-44-6148-01	TCGA-50-5055-01	TCGA-55-7570-01	TCGA-55-A494-01	TCGA-73-4659-01	TCGA-78-8662-01	TCGA-93-A4JO-01	TCGA-L9-A8F4-01
TCGA-44-3398-11	TCGA-05-4402-01	TCGA-44-6774-01	TCGA-50-5066-01	TCGA-55-7573-01	TCGA-55-A4DF-01	TCGA-73-4662-01	TCGA-80-5607-01	TCGA-93-A4JP-01	TCGA-MN-A4N1-01
TCGA-44-5645-11	TCGA-05-4403-01	TCGA-44-6775-01	TCGA-50-5068-01	TCGA-55-7574-01	TCGA-55-A4DG-01	TCGA-73-4666-01	TCGA-80-5608-01	TCGA-93-A4JQ-01	TCGA-MN-A4N4-01
TCGA-44-6144-11	TCGA-05-4405-01	TCGA-44-6776-01	TCGA-50-5072-01	TCGA-55-7576-01	TCGA-55-A57B-01	TCGA-73-4668-01	TCGA-80-5611-01	TCGA-95-7039-01	TCGA-MN-A4N5-01
TCGA-44-6145-11	TCGA-05-4410-01	TCGA-44-6777-01	TCGA-50-5930-01	TCGA-55-7724-01	TCGA-62-8394-01	TCGA-73-4670-01	TCGA-83-5908-01	TCGA-95-7043-01	TCGA-MP-A4SV-01
TCGA-44-6146-11	TCGA-05-4415-01	TCGA-44-6778-01	TCGA-50-5931-01	TCGA-55-7725-01	TCGA-62-8395-01	TCGA-73-4675-01	TCGA-86-6562-01	TCGA-95-7562-01	TCGA-MP-A4SW-01
TCGA-44-6147-11	TCGA-05-4417-01	TCGA-44-6779-01	TCGA-50-5932-01	TCGA-55-7726-01	TCGA-62-8397-01	TCGA-73-4676-01	TCGA-86-6851-01	TCGA-95-7567-01	TCGA-MP-A4SY-01
TCGA-44-6148-11	TCGA-05-4418-01	TCGA-44-7659-01	TCGA-50-5933-01	TCGA-55-7727-01	TCGA-62-8398-01	TCGA-73-4677-01	TCGA-86-7701-01	TCGA-95-7944-01	TCGA-MP-A4T4-01
TCGA-44-6776-11	TCGA-05-4420-01	TCGA-44-7660-01	TCGA-50-5935-01	TCGA-55-7728-01	TCGA-62-8399-01	TCGA-73-7498-01	TCGA-86-7711-01	TCGA-95-7947-01	TCGA-MP-A4T6-01
TCGA-44-6777-11	TCGA-05-4422-01	TCGA-44-7661-01	TCGA-50-5936-01	TCGA-55-7815-01	TCGA-62-8402-01	TCGA-73-7499-01	TCGA-86-7713-01	TCGA-95-7948-01	TCGA-MP-A4T7-01
TCGA-44-6778-11	TCGA-05-4424-01	TCGA-44-7662-01	TCGA-50-5939-01	TCGA-55-7816-01	TCGA-62-A46O-01	TCGA-73-A9RS-01	TCGA-86-7714-01	TCGA-95-8039-01	TCGA-MP-A4T8-01
TCGA-49-4490-11	TCGA-05-4425-01	TCGA-44-7667-01	TCGA-50-5941-01	TCGA-55-7903-01	TCGA-62-A46P-01	TCGA-75-5122-01	TCGA-86-7953-01	TCGA-95-8494-01	TCGA-MP-A4T9-01
TCGA-49-4512-11	TCGA-05-4426-01	TCGA-44-7669-01	TCGA-50-5942-01	TCGA-55-7907-01	TCGA-62-A46R-01	TCGA-75-5125-01	TCGA-86-7954-01	TCGA-95-A4VK-01	TCGA-MP-A4TA-01
TCGA-49-6742-11	TCGA-05-4427-01	TCGA-44-7670-01	TCGA-50-5944-01	TCGA-55-7910-01	TCGA-62-A46S-01	TCGA-75-5126-01	TCGA-86-7955-01	TCGA-95-A4VN-01	TCGA-MP-A4TC-01
TCGA-49-6743-11	TCGA-05-4430-01	TCGA-44-7671-01	TCGA-50-5946-01	TCGA-55-7911-01	TCGA-62-A46U-01	TCGA-75-5146-01	TCGA-86-8054-01	TCGA-95-A4VP-01	TCGA-MP-A4TD-01
TCGA-49-6744-11	TCGA-05-4432-01	TCGA-44-7672-01	TCGA-50-6590-01	TCGA-55-7913-01	TCGA-62-A46V-01	TCGA-75-5147-01	TCGA-86-8055-01	TCGA-97-7546-01	TCGA-MP-A4TE-01
TCGA-49-6745-11	TCGA-05-4433-01	TCGA-44-8117-01	TCGA-50-6591-01	TCGA-55-7914-01	TCGA-62-A46Y-01	TCGA-75-6203-01	TCGA-86-8056-01	TCGA-97-7547-01	TCGA-MP-A4TF-01
TCGA-49-6761-11	TCGA-05-4434-01	TCGA-44-8119-01	TCGA-50-6592-01	TCGA-55-7994-01	TCGA-62-A470-01	TCGA-75-6205-01	TCGA-86-8073-01	TCGA-97-7552-01	TCGA-MP-A4TH-01
TCGA-50-5930-11	TCGA-05-5420-01	TCGA-44-8120-01	TCGA-50-6593-01	TCGA-55-7995-01	TCGA-62-A471-01	TCGA-75-6206-01	TCGA-86-8074-01	TCGA-97-7553-01	TCGA-MP-A4TI-01
TCGA-50-5931-11	TCGA-05-5423-01	TCGA-44-A479-01	TCGA-50-6594-01	TCGA-55-8085-01	TCGA-62-A472-01	TCGA-75-6207-01	TCGA-86-8075-01	TCGA-97-7554-01	TCGA-MP-A4TJ-01
TCGA-50-5932-11	TCGA-05-5425-01	TCGA-44-A47A-01	TCGA-50-6595-01	TCGA-55-8087-01	TCGA-64-1676-01	TCGA-75-6211-01	TCGA-86-8076-01	TCGA-97-7937-01	TCGA-MP-A4TK-01
TCGA-50-5933-11	TCGA-05-5428-01	TCGA-44-A47B-01	TCGA-50-6597-01	TCGA-55-8089-01	TCGA-64-1677-01	TCGA-75-6212-01	TCGA-86-8278-01	TCGA-97-7938-01	TCGA-MP-A5C7-01
TCGA-50-5935-11	TCGA-05-5429-01	TCGA-44-A47G-01	TCGA-50-6673-01	TCGA-55-8090-01	TCGA-64-1678-01	TCGA-75-6214-01	TCGA-86-8279-01	TCGA-97-7941-01	TCGA-NJ-A4YF-01
TCGA-50-5936-11	TCGA-05-5715-01	TCGA-44-A4SS-01	TCGA-50-7109-01	TCGA-55-8091-01	TCGA-64-1679-01	TCGA-75-7025-01	TCGA-86-8280-01	TCGA-97-8171-01	TCGA-NJ-A4YG-01
TCGA-50-5939-11	TCGA-35-3615-01	TCGA-44-A4SU-01	TCGA-50-8457-01	TCGA-55-8092-01	TCGA-64-1680-01	TCGA-75-7027-01	TCGA-86-8281-01	TCGA-97-8172-01	TCGA-NJ-A4YI-01
TCGA-50-6595-11	TCGA-35-4122-01	TCGA-49-4486-01	TCGA-50-8459-01	TCGA-55-8094-01	TCGA-64-1681-01	TCGA-75-7030-01	TCGA-86-8358-01	TCGA-97-8174-01	TCGA-NJ-A4YP-01
TCGA-55-6968-11	TCGA-35-4123-01	TCGA-49-4487-01	TCGA-50-8460-01	TCGA-55-8096-01	TCGA-64-5774-01	TCGA-75-7031-01	TCGA-86-8359-01	TCGA-97-8175-01	TCGA-NJ-A4YQ-01
TCGA-55-6969-11	TCGA-35-5375-01	TCGA-49-4488-01	TCGA-53-7624-01	TCGA-55-8097-01	TCGA-64-5775-01	TCGA-78-7143-01	TCGA-86-8585-01	TCGA-97-8176-01	TCGA-NJ-A55A-01
TCGA-55-6970-11	TCGA-38-4625-01	TCGA-49-4490-01	TCGA-53-7626-01	TCGA-55-8203-01	TCGA-64-5778-01	TCGA-78-7145-01	TCGA-86-8668-01	TCGA-97-8177-01	TCGA-NJ-A55O-01
TCGA-55-6971-11	TCGA-38-4626-01	TCGA-49-4494-01	TCGA-53-7813-01	TCGA-55-8204-01	TCGA-64-5779-01	TCGA-78-7146-01	TCGA-86-8669-01	TCGA-97-8179-01	TCGA-NJ-A55R-01
TCGA-55-6972-11	TCGA-38-4627-01	TCGA-49-4501-01	TCGA-53-A4EZ-01	TCGA-55-8205-01	TCGA-64-5781-01	TCGA-78-7147-01	TCGA-86-8671-01	TCGA-97-8547-01	TCGA-NJ-A7XG-01
TCGA-55-6975-11	TCGA-38-4628-01	TCGA-49-4505-01	TCGA-55-1592-01	TCGA-55-8206-01	TCGA-64-5815-01	TCGA-78-7148-01	TCGA-86-8672-01	TCGA-97-8552-01	TCGA-O1-A52J-01
TCGA-55-6978-11	TCGA-38-4629-01	TCGA-49-4506-01	TCGA-55-1594-01	TCGA-55-8207-01	TCGA-67-3770-01	TCGA-78-7149-01	TCGA-86-8673-01	TCGA-97-A4LX-01	TCGA-S2-AA1A-01
TCGA-55-6979-11	TCGA-38-4630-01	TCGA-49-4507-01	TCGA-55-1595-01	TCGA-55-8208-01	TCGA-67-3771-01	TCGA-78-7150-01	TCGA-86-8674-01	TCGA-97-A4M0-01	
TCGA-55-6980-11	TCGA-38-4631-01	TCGA-49-4510-01	TCGA-55-1596-01	TCGA-55-8299-01	TCGA-67-3772-01	TCGA-78-7152-01	TCGA-86-A456-01	TCGA-97-A4M1-01	
TCGA-55-6981-11	TCGA-38-4632-01	TCGA-49-4512-01	TCGA-55-5899-01	TCGA-55-8301-01	TCGA-67-3773-01	TCGA-78-7153-01	TCGA-86-A4D0-01	TCGA-97-A4M2-01	
TCGA-55-6982-11	TCGA-38-6178-01	TCGA-49-4514-01	TCGA-55-6543-01	TCGA-55-8302-01	TCGA-67-3774-01	TCGA-78-7154-01	TCGA-86-A4JF-01	TCGA-97-A4M3-01	
TCGA-55-6983-11	TCGA-38-7271-01	TCGA-49-6742-01	TCGA-55-6642-01	TCGA-55-8505-01	TCGA-67-4679-01	TCGA-78-7155-01	TCGA-86-A4P7-01	TCGA-97-A4M5-01	
TCGA-55-6984-11	TCGA-38-A44F-01	TCGA-49-6743-01	TCGA-55-6712-01	TCGA-55-8506-01	TCGA-67-6215-01	TCGA-78-7156-01	TCGA-86-A4P8-01	TCGA-97-A4M6-01	
TCGA-55-6985-11	TCGA-44-2655-01	TCGA-49-6744-01	TCGA-55-6968-01	TCGA-55-8507-01	TCGA-67-6216-01	TCGA-78-7158-01	TCGA-91-6828-01	TCGA-97-A4M7-01	
TCGA-55-6986-11	TCGA-44-2656-01	TCGA-49-6745-01	TCGA-55-6969-01	TCGA-55-8508-01	TCGA-67-6217-01	TCGA-78-7159-01	TCGA-91-6829-01	TCGA-99-7458-01	
TCGA-73-4676-11	TCGA-44-2657-01	TCGA-49-6761-01	TCGA-55-6970-01	TCGA-55-8510-01	TCGA-69-7760-01	TCGA-78-7160-01	TCGA-91-6830-01	TCGA-99-8025-01	
TCGA-91-6828-11	TCGA-44-2659-01	TCGA-49-6767-01	TCGA-55-6971-01	TCGA-55-8511-01	TCGA-69-7761-01	TCGA-78-7161-01	TCGA-91-6831-01	TCGA-99-8028-01	
TCGA-91-6829-11	TCGA-44-2661-01	TCGA-49-AAQV-01	TCGA-55-6972-01	TCGA-55-8512-01	TCGA-69-7763-01	TCGA-78-7162-01	TCGA-91-6835-01	TCGA-99-8032-01	
TCGA-91-6831-11	TCGA-44-2662-01	TCGA-49-AAR0-01	TCGA-55-6975-01	TCGA-55-8513-01	TCGA-69-7764-01	TCGA-78-7163-01	TCGA-91-6836-01	TCGA-99-8033-01	
TCGA-91-6835-11	TCGA-44-2665-01	TCGA-49-AAR2-01	TCGA-55-6978-01	TCGA-55-8514-01	TCGA-69-7765-01	TCGA-78-7166-01	TCGA-91-6840-01	TCGA-99-AA5R-01	
TCGA-91-6836-11	TCGA-44-2666-01	TCGA-49-AAR3-01	TCGA-55-6979-01	TCGA-55-8614-01	TCGA-69-7973-01	TCGA-78-7167-01	TCGA-91-6847-01	TCGA-J2-8192-01	
TCGA-91-6847-11	TCGA-44-2668-01	TCGA-49-AAR4-01	TCGA-55-6980-01	TCGA-55-8615-01	TCGA-69-7974-01	TCGA-78-7220-01	TCGA-91-6848-01	TCGA-J2-8194-01	
TCGA-91-6849-11	TCGA-44-3396-01	TCGA-49-AAR9-01	TCGA-55-6981-01	TCGA-55-8616-01	TCGA-69-7978-01	TCGA-78-7535-01	TCGA-91-6849-01	TCGA-J2-A4AD-01	
