# Supplementary material for: Characterization of the Oncogenic Potential of Eukaryotic Initiation Factor 4A1 in Lung Adenocarcinoma via Cell Cycle Regulation and Immune Microenvironment Reprogramming
Source: Biology (Basel). 2022 Jun 28;11(7):975. doi: 10.3390/biology11070975 (PMC9311917; doi:10.3390/biology11070975)
Supplement: Supplementary file 1 [file biology-11-00975-s001.zip › biology-1772343-supplementary-Tables S1 and S2.pdf]

Table S1. The gene lists of positive correlation with EIF4A1

Gene	r	Gene	r	Gene	r	Gene	r
EIF5AL1	0.75	WRAP53	0.46	RPL7L1	0.43	NOL10	0.41
TSR1	0.71	GSG2	0.46	PAK1IP1	0.43	FARSB	0.41
C1QBP	0.69	PA2G4	0.46	PPIL1	0.42	PWP1	0.41
SCO1	0.66	CSE1L	0.45	DBF4	0.42	CDC25A	0.41
EIF5A	0.66	URB2	0.45	PPAT	0.42	C11orf82	0.41
SENP3	0.66	RPP40	0.45	ATIC	0.42	UHRF1	0.41
YWHAE	0.62	EEF1E1	0.45	XPO5	0.42	C10orf119	0.41
NUP88	0.61	FAM64A	0.45	DDX18	0.42	THOC4	0.41
PHF23	0.6	CDCA5	0.45	CCNA2	0.42	UNG	0.4
ELAC2	0.55	MRPL44	0.45	CHEK1	0.42	DNAJC9	0.4
AURKB	0.54	HSPD1	0.45	PRPF40A	0.42	RRM2	0.4
COX10	0.54	EIF3J	0.44	NCAPD2	0.42	EIF2S2	0.4
FXR2	0.54	NOL7	0.44	NUDCD1	0.42	KPNA2	0.4
DHX33	0.54	TMEM93	0.44	NDUFS1	0.42	GEMIN4	0.4
PFN1	0.53	SRPK1	0.44	CENPH	0.42	NUP153	0.4
COPS3	0.53	CCT4	0.44	CENPO	0.42	ZNF286A	0.4
UBE2G1	0.53	MCM4	0.44	MCM10	0.42	DTL	0.4
WDR43	0.52	ABCE1	0.44	NOP56	0.42	NUDT15	0.4
PSMB6	0.51	SSRP1	0.44	PLK1	0.42	GRPEL1	0.4
MYBBP1A	0.5	MAD2L1	0.44	BUB1B	0.42	PRPF8	0.4
CCNB1	0.5	PELP1	0.44	PUS7	0.41	WDR75	0.4
DDX21	0.49	MRPL19	0.44	CDKN3	0.41	PRKDC	0.4
LYAR	0.49	PRR11	0.44	DSCC1	0.41	CENPE	0.4
DARS2	0.49	NLE1	0.44	TEX10	0.41	UTP18	0.4
WDR12	0.49	PGAM5	0.44	STRAP	0.41	SLC25A11	0.4
CCT7	0.48	OLA1	0.44	GABPB1	0.41	DDX10	0.4
TIMM22	0.48	C10orf2	0.44	BCCIP	0.41	MRPL42	0.4
PAICS	0.48	SNRPD1	0.44	CRK	0.41	MTHFD2	0.4
MAP2K4	0.48	NCL	0.43	HMGA1	0.41	CCDC86	0.4
RIOK1	0.48	GLOD4	0.43	GNL3	0.41	NCAPH	0.4
BUB3	0.48	PFAS	0.43	NOP58	0.41	DCAF13	0.4
DVL2	0.48	NCAPG	0.43	LRPPRC	0.41	AHCY	0.4
RNMTL1	0.48	MIS12	0.43	NLN	0.41	HNRNPC	0.4
NOP14	0.48	RPL26	0.43	RABEP1	0.41	PNO1	0.4
RAN	0.47	YWHAQ	0.43	CCT8	0.41	VDAC2	0.4
RPA1	0.47	NUP37	0.43	FOXM1	0.41	ACLY	0.4
NOLC1	0.47	MPDU1	0.43	DKC1	0.41	KPNB1	0.4
CCT3	0.46	OVCA2	0.43	UCHL5	0.41	NOL11	0.4
MKI67IP	0.46	DENR	0.43	DARS	0.41	STIP1	0.4
DULLARD	0.46	EIF2S1	0.43	XRCC5	0.41	CCT5	0.4

Table S2. The gene lists of negative correlation with EIF4A1

Gene	r	Gene	r	Gene	r	Gene	r
CCDC159	-0.38	UBA7	-0.32	ABCC6	-0.31	RPS6KA1	-0.3
LMF1	-0.37	FAAH	-0.32	HLA-DOB	-0.31	C5orf45	-0.3
CBX7	-0.36	CTSH	-0.32	PIGQ	-0.31	AGXT2L2	-0.3
TNFRSF14	-0.35	CD74	-0.32	SFI1	-0.31	TUBGCP6	-0.3
ZMAT1	-0.34	C1orf63	-0.32	HLA-DPB1	-0.31	FAM193B	-0.3
HLA-DMA	-0.34	FBXO44	-0.32	HLA-DRB1	-0.31	KLHDC1	-0.3
ECHDC2	-0.34	KLHDC7A	-0.32	LDLRAP1	-0.31	DAPK2	-0.3
PIK3IP1	-0.34	FUCA1	-0.32	CCDC88B	-0.31	CHKB-CPT1B	-0.3
C21orf2	-0.33	GGT3P	-0.31	GNPTG	-0.31	LOC115110	-0.3
ZNF688	-0.33	TMEM125	-0.31	NAPSB	-0.3	TTLL3	-0.3
SEPW1	-0.32	TMEM219	-0.31	FMO4	-0.3		
CCDC159	-0.38	C17orf108	-0.31	POU6F1	-0.3		
